# Micro/nanoplastics–metal(loid) co-exposure in rice: evidence, mechanisms, and food-safety implications

**DOI:** 10.3389/fpls.2026.1963280

**Published:** 2026-10-20

**Authors:** Tiansheng Liu, Wenjia Li, Munan Tang, Yuran Wang, Jianyu Wang, Yuxiang Zhang, Zunqi Liu

**Affiliations:** 1 College of Landscape Architecture, Changchun University, Changchun, China; 2 Agronomy College, Shenyang Agricultural University, Shenyang, China; 3 Key Laboratory of Biochar and Soil Improvement, Ministry of Agriculture and Rural Affairs, Shenyang, China

**Keywords:** context-dependent responses, grain food safety, integrated management, metal(loid)s, micro/nanoplastics, rice agroecosystems

## Abstract

Micro/nanoplastics (MNPs) and metal(loid)s frequently co-occur in rice agroecosystems, where legacy metal(loid) contamination and continuing plastic inputs may jointly threaten soil quality and rice safety. Co-exposure complicates risk assessment because rice responses cannot always be predicted from MNP-only or metal(loid)-only treatments. To address this, the review integrates mechanistic findings into a mixture- and context-dependent synthesis linking MNP traits and metal(loid) identity/speciation to soil–rhizosphere processes, plant uptake/partitioning, and grain food-safety outcomes. Metal(loid) exposure can disrupt growth by altering metal(loid) uptake, nutrient homeostasis, and redox regulation, whereas MNPs can modify root development, soil–root interfacial processes, and microbially mediated metal(loid) cycling. Consequently, co-exposure may generate responses with directions and magnitudes that depend on metal(loid) speciation, bioavailability, tissue partitioning, and plant performance, varying with metal(loid) identity, MNP properties, exposure dose, and environmental conditions. This review synthesizes current evidence on soil and rhizosphere processes, root uptake and internal transport, plant responses, and grain food-safety implications, with emphasis on Cd, inorganic As, Hg/MeHg, and Pb. It further identifies key evidence gaps, particularly the limited mature-grain and field-based evidence, and proposes integrated management strategies that reduce metal(loid) bioavailability and concentrations in mature rice grain, while avoiding increased MNP burdens, yield penalties, or deterioration in grain quality.

## 1 Introduction

Micro/nanoplastics (MNPs) are persistent co-contaminants in rice agroecosystems, where they commonly co-occur with trace metal(loid)s and nutrient pollutants rather than appearing as single-stressor exposures. Such multi-contaminant exposure is evident in large river basins, such as the Yangtze River Economic Belt: even with policy-driven declines in point-source metal(loid)s emissions, legacy contamination in sediments and diffuse agricultural inputs continue to expose rice and linked water compartments to metal(loid)s such as cadmium (Cd) and arsenic (As), while ongoing microplastic flux adds further pressure on downstream ecosystems (Cao and Wang, 2026). Multiple co-existing contaminant inputs can jointly affect soil functions, making multi-stressor exposure a realistic scenario for food production. Evidence from coastal agricultural soils further supports this co-occurrence, with plastic mulching both increasing microplastic loads and altering trace element behavior—raising concerns for long-term soil health and food safety and motivating integrated ecological toxicology approaches (Ashwathi et al., 2025).

For rice, metal(loid)s and MNPs converge on measurable phenotypic and elemental endpoints, although they act through distinct but overlapping response pathways. Metal(loid)s can disrupt rice performance by altering uptake and redistribution, including transporter-mediated transport and internal partitioning, perturbing nutrient homeostasis, and activating cellular stress-response and repair processes (Kaur et al., 2021; Mao et al., 2022; Qadeer et al., 2026). In parallel, MNPs have been reported to affect growth and photosynthesis through changes in root development and early establishment, soil physicochemical properties, and rhizosphere processes that regulate the bioavailability and transport of co-present metal(loid)s (Tang et al., 2022; Deng et al., 2025; Moharana et al., 2025). Beyond short-term physiology, combined exposures may further influence grain-related outcomes and food-safety risk by altering the plant metallome and element partitioning among root, shoot, and edible tissues, alongside shifts in soil microbial functions linked to carbon, nitrogen, and sulfur cycling (Xie et al., 2025).

Although rice responses to metal(loid)s or to MNPs alone are well documented, evidence on their combined effects remains limited (Moharana et al., 2025; Xie et al., 2025). Co-occurrence does not necessarily produce responses that can be inferred from single-stressor treatments. Instead, the direction and magnitude of co-exposure responses may vary with metal(loid) identity and chemical speciation, polymer type, particle size, surface characteristics, exposure concentration, and environmental conditions (Liu et al., 2022). Here, “context-dependent responses” means that the direction and magnitude of co-exposure effects cannot be reliably predicted from single-stressor (MNP-only or metal(loid)-only) experiments, because metal(loid) speciation/bioavailability and plant uptake/partitioning are jointly modulated by MNP traits, exposure dose, and environmental/soil conditions. This variability suggests that co-exposure outcomes may reflect coupled mechanisms among (i) plastic-driven changes in metal(loid)s bioavailability/speciation and transport, (ii) rice physiological signaling and antioxidant/repair responses, (iii) root–shoot divergence and stage-dependent sensitivity, and (iv) downstream impacts on tissue element accumulation and food-safety relevance, together with longer-term effects on soil microbiomes and production outcomes. However, most existing studies rely on simplified single-pollutant designs, and comprehensive syntheses linking pollutant physicochemical traits to the full chain of rice responses across biological and temporal scales remain scarce (Gui et al., 2025; Lin et al., 2025; Cao and Wang, 2026). However, these limitations also constrain interpretation: most syntheses do not critically map how specific pollutant physicochemical determinants translate into a coherent mechanistic chain from soil–rhizosphere processes to rice uptake/transport and stage-specific tissue partitioning, and then to mature-grain food-safety endpoints under mixture- and field-relevant conditions. As a result, key knowledge gaps remain regarding which coupling steps (metal(loid) bioavailability/speciation, root-to-shoot signaling, or downstream tissue accumulation) dominate mixture effects and how these translate into risk-relevant outcomes for grains.

Accordingly, this review consolidates evidence on MNP–metal(loid) co-contamination in rice, summarizes mechanisms and determinants of co-exposure responses that vary with metal(loid) identity/speciation and exposure conditions, and outlines potential management routes—covering system-level and basin-scale governance, field-scale practices aimed at stabilizing metal(loid)s bioavailability and limiting plastics-mediated transformations, and plant/rhizosphere strategies that may reduce metal(loid)s entry flux and reshape detoxification and tissue partitioning—with the aim of reducing metal(loid) bioavailability and grain-related food-safety risks while avoiding the displacement of environmental burdens across compartments or over time.

## 2 Literature search

Relevant studies were retrieved from the Web of Science Core Collection and PubMed databases from inception to August 2026. The search strategy used the following terms: (microplastic OR nanoplastic OR “plastic particle” OR “plastic debris” OR polystyrene OR polyethylene OR polypropylene OR polyvinyl chloride OR polyethylene terephthalate) AND (rice OR “Oryza sativa”). The retrieved records were then manually screened to identify original English-language studies investigating rice or rice soil–plant systems co-exposed to MNPs and at least one metal(loid). Studies examining MNPs or metal(loid)s alone were excluded. The screening workflow is summarized in **Supplementary Figure 1**, including the numbers of records identified, duplicates removed, records excluded after title/abstract screening, full-text reports assessed for eligibility, and full-text exclusions with reasons. This approach enabled a focused assessment of interaction-related effects on rice growth, physiological responses, metal(loid) uptake, rhizosphere processes, and potential grain-safety risks.

## 3 Phenotypic and elemental endpoints in rice under MNP–metal(loid) co-exposure

### 3.1 Growth and root–shoot development

Emerging evidence suggests that MNP–metal(loid) co-exposure in rice can produce growth and root–shoot responses that are not readily predicted from metal(loid)s-only or MNP-only treatments (Jiang M. et al., 2024; Xu et al., 2025; Liu et al., 2026). These endpoint patterns indicate that developmental responses under co-exposure may differ from those observed under either single-stressor treatment; however, formal interaction classification requires appropriate single-stressor controls and an explicit additive null model (Liu et al., 2022; Liu et al., 2026). Therefore, differences in growth, biomass, or tissue metal(loid) concentrations should not alone be interpreted as evidence of synergistic or antagonistic interactions. Notably, opposite outcomes (enhanced vs reduced metal(loid) uptake/accumulation) have been reported across studies. This inconsistency is largely attributable to (i) different MNP physicochemical properties that modulate metal(loid) speciation and bioavailability, (ii) differences in exposure dose and timing that alter root–shoot developmental sensitivity, (iii) distinct exposure media (hydroponics vs soils) that change sorption/transport conditions in the rhizosphere, and (iv) stage- and organ-specific partitioning, which can decouple growth/physiology endpoints from tissue metal(loid) loading. In addition, formal classification of synergy/antagonism requires appropriate single-stressor controls and an explicit additive null model; therefore, reported directionality should be interpreted as variable rather than automatically indicating a universal interaction type.

Across metal(loid)s, development-linked endpoints relevant to growth and yield often vary with the coupled exposure conditions. For Cd, hydroponic studies often report dose-dependent changes in root-establishment endpoints under co-exposure: either Cd or NPs alone typically impair root development (e.g., root length, root tips, and root surface area), but co-exposure can be associated with different response directions across MNP doses: low MNP loads may coincide with less severe Cd-related root damage, whereas higher MNP loads may be associated with stronger inhibition (Liu et al., 2022; Liu et al., 2026). Comparable shifts have been reported in Cd co-exposed with MNPs of different polymer identities, where co-exposure can shift not only effect magnitude but also tissue- and stage-dependent bias (from broad rhizosphere–plant suppression to preferential impairment of root-related endpoints), suggesting that polymer-specific properties alter where interaction effects emerge along the root–shoot trajectory (Jiang M. et al., 2024; Xu et al., 2025; Gao et al., 2026; Zhou et al., 2026). For As–MNP co-exposure, non-equivalent outcomes are also frequently reported, with stronger inhibition often occurring in aboveground growth (e.g., biomass reduction) while root and leaf endpoints may not change in parallel; this organ-level desynchronization supports departures from additive expectations and divergence in root–shoot allocation (Mamathaxim et al., 2023; Xu C. et al., 2023; Wu et al., 2025a; Xu, 2026). Under Cu exposure, microplastics (MPs) may decouple growth responses from internal metal(loid)s metal(loid)s loading in a polymer-dependent manner. PP MPs partly improved shoot growth but increased seedling Cu accumulation, whereas PS MPs lowered Cu bioavailability and tissue accumulation without restoring POD activity (Jin et al., 2024; Hao et al., 2026). This relation may shift at the germination stage: low-dose PS MPs reduced seedling Cu accumulation by 34% and slightly increased the Cu EC50, apparently through association with the seed coat rather than substantial Cu adsorption, alongside lower ROS and enhanced CAT and GSH responses (Liu et al., 2024). Thus, apparent improvement in shoot growth may coexist with greater internal metal(loid)s loading and contrasting root–shoot antioxidant responses. Across configurations, these findings indicate that growth disruptions stem from coordinated reorganization of developmental processes driven by MNPs–metal(loid)s interactions, making integrated management (e.g., combined remediation strategies) more likely to restore growth at the system level than to produce only compensatory shifts expected from single-pollutant responses (Irshad et al., 2024; Xu, 2026; Yu et al., 2026).

### 3.2 Leaf photosynthetic performance and gas-exchange function

In rice, co-exposure more often produces disturbances that are not reproduced by combining metal(loid)-only and plastic-only outcomes—that is, co-exposure effects on leaf photosynthetic capacity and gas exchange that are not reproduced by simply combining metal(loid)-only and plastic-only outcomes—than either stressor alone. Leaf responses therefore commonly show departures from additive expectations (e.g., antagonistic or synergistic patterns) and vary with the coupled exposure context, making leaves a sensitive “signal layer” for mixture effects (Dong et al., 2020; Irshad et al., 2024). Across shared endpoints, co-exposure tends to show declines in chlorophyll and suppression of photosynthesis-related processes (e.g., altered chlorophyll fluorescence and reduced net photosynthetic rate) (Jinhua et al., 2026; Noor et al., 2026). Gas exchange changes in parallel—often with reduced stomatal conductance (Gs) and transpiration rate (Tr), alongside corresponding shifts in intercellular CO_2_ (Ci)—supporting the view that leaf function under mixture exposure reflects functional remodeling, not overall stress intensity (Dong et al., 2020; Irshad et al., 2024).

For As–MNPs, hydroponic studies indicate that co-exposure (e.g., As(III) with microplastics such as PS or PTFE) inhibits rice leaf photosynthesis, with reduced net photosynthetic rate, altered chlorophyll fluorescence indices, and decreased chlorophyll a; inhibition is often stronger and dose-dependent versus As alone, with some photosynthetic impairments attenuating at lower plastic loads but intensifying as plastic exposure increases (Dong et al., 2020). Transcriptomic evidence further supports leaf-level reprogramming rather than additivity. Similar patterns occur in Pb–MPs co-exposure, where Pn decreases together with reduced Gs and Tr and altered Ci. Recovery studies further indicate that biochar plus melatonin can improve Pn, Tr, Gs, Ci, and oxidative-stress indicators under Pb–MP co-exposure, although these improvements should not be interpreted as evidence of a synergistic interaction without formal interaction testing (Du et al., 2026; Peng et al., 2026). For Cd–MPs, direct gas-exchange data may be limited, but combined-exposure and mitigation studies fit the same framework: mixture-condition interventions (e.g., biochar + melatonin) improve chlorophyll and leaf water/antioxidant status and reduce Cd-related translocation/accumulation trends (Yang et al., 2026). Overall, leaf photosynthetic performance and gas exchange provide sensitive endpoints of MNP–metal(loid) co-exposure in rice, including chlorophyll decline, photosynthetic and fluorescence anomalies, and coordinated Gs/Tr/Ci shifts. Their direction and magnitude are conditional on the exposure system and should not be inferred from metal(loid)s -only treatments.

### 3.3 Yield and grain-production-related outcomes

In rice, MNPs–metal(loid) co-exposure can alter yield formation and grain-production traits, including biomass, grain yield, and yield-related phenotypes. Yield-related outcomes under co-exposure cannot be assumed from the separate effects of metal(loid)-only and MNPs-only treatments. Under co-stress, yield outcomes often reflect altered damage–recovery linkages—i.e., the stress-to-yield pathway and the conditions required for recovery depend on the specific co-exposure setting—so the magnitude of recovery may vary accordingly and should not be inferred from the separate effects of MNPs and metal(loid)s (Xu C. et al., 2023; Du et al., 2026; Noor et al., 2026; Yang et al., 2026).

For example, in the Pb–microplastics system, co-exposure markedly suppresses yield-related endpoints: relative to control, Pb + MPs (1%) reduces biomass (BY) by 25.26% and grain yield (GY) by 41.51%. Targeted mitigation was observed in the Pb–MP co-exposure system: biochar plus melatonin increased BY 18.20% and GY by 39.59% relative to Pb–MP co-exposure alone, together with enhanced antioxidant defense and improved soil fertility-related properties (Du et al., 2026). Comparable findings occur in Cd–MPs co-exposure, where yield losses are often substantial (e.g., reductions on the order of ~81% under Cd + MPs) (Jinhua et al., 2026). In the reported Cd–MP co-exposure system, nano-modified biochar improved yield-related outcomes while reducing soil Cd bioavailability and Cd accumulation in roots and aboveground tissues by lowering soil Cd bioavailability and Cd accumulation in roots and aboveground tissues, and by upregulating genes linked to osmotic adjustment and antioxidant defense; Cd–MPs plus Si can similarly shift outcomes upward relative to the ~40% decrease seen under Cd–MPs, coinciding with improved chlorophyll/antioxidant status and downregulation of Cd uptake-related genes (Noor et al., 2026). Overall, both the severity of yield decline and the capacity for recovery under co-exposure are strongly conditioned by the mixture context rather than following linear extrapolations from Cd-only or MNPs-only treatments.

### 3.4 Tissue element accumulation, partitioning, and food-safety relevance

Under MNP–metal(loid) co-exposure, tissue accumulation and partitioning cannot be reliably inferred from single-stressor results. Evidence indicates that co-exposure can alter root–shoot–grain/seed partitioning of Cd and As. However, food-safety relevance should be evaluated primarily from mature-grain concentration, chemical speciation, and bioaccessibility rather than from root, seedling, or vegetative-tissue measurements alone (An et al., 2025a; An et al., 2025b; Jinhua et al., 2026; Xu, 2026). To translate measured metal(loid) concentrations in mature rice into food-safety relevance, findings should be interpreted against international or regional maximum limits for key contaminants (e.g., Cd, inorganic As, Pb, and Hg where applicable) (Authority, 2009). Human dietary exposure depends on both consumption level and gastrointestinal bioavailability, so grain concentration alone cannot fully capture risk; risk implications should therefore be evaluated using estimated dietary intake (concentration × consumption) and, where feasible, bioaccessibility- or speciation-informed estimates (Brandon et al., 2014). Therefore, food-safety implications under co-exposure depend on observed accumulation, chemical speciation, bioaccessibility, and tissue distribution, particularly in mature grains, rather than on extrapolation from individual-pollutant studies.

For Cd, some co-exposure studies report increased accumulation in edible tissues and altered partitioning among roots, shoots, and grains relative to Cd-only exposure (Gao et al., 2026). In paddy systems, Cd reactivation from fixed states differs by polymer type (e.g., PET vs. PVC), leading to polymer-dependent changes in grain Cd concentration relative to Cd-only exposure—for instance, under a PET residual co-exposure context, brown rice Cd increases from 0.03 mg/kg to 0.06 mg/kg (Zhou et al., 2026). Multi-tissue data further support non-extrapolable behavior: when Cd is 5 mg/kg and mPE is 1%, Cd accumulation in roots, stems, and seeds all increases, unlike Cd-only treatments that show more limited and less coordinated across-tissue rises (Gao et al., 2026). Importantly, grain Cd concentration may not fully represent dietary exposure risk when MPs are co-ingested. In mice fed Cd-contaminated rice, dietary PE, PS, PP, or PA MPs (2 μg g^−1^) increased tissue Cd accumulation by 1.17–1.38-fold. PE-associated changes in gut microbiota and metabolites were linked to greater intestinal Cd solubility and transport (Chen et al., 2024). Thus, rice-based risk assessment should distinguish grain Cd loading from its potential gastrointestinal bioavailability under MP co-exposure. Nevertheless, the magnitude of co-ingestion-related risk in humans remains uncertain because existing evidence is largely derived from preclinical studies and does not yet cover the full range of realistic particle characteristics and exposure conditions (Kogut et al., 2026). Intervention evidence is consistent: biochar plus melatonin can reduce soil Cd bioavailability and decrease Cd accumulation in rice under co-exposure, indicating that food-safety endpoints can be reshaped through strategies tailored to the mixture context (Yang et al., 2026). For As, co-exposure alters not only effect magnitude but also transport routes and partitioning within edible tissues. Under MNP–As co-exposure, As accumulation can vary in direction and magnitude; under certain nano-plastic co-exposure conditions, As increases in roots and shoots by 24.5% and 70.9% relative to As-only, consistent with greater root-to-shoot As translocation under the tested nanoplastic co-exposure conditions. Polymer type and particle size/shape strongly modulate partitioning, and different PLA size classes can therefore produce directionally different responses in As uptake and translocation. Risk assessment should consequently be grounded in material characteristics and the specific co-exposure context rather than relying solely on As-only trend patterns (Xu C. et al., 2023; Jiang Y. et al., 2024; An et al., 2025b). Food-safety assessment should also consider market-relevant quality. The impacts of MNP–metal(loid)s co-exposure on fragrant-rice aroma, including 2-AP biosynthesis, remain a major evidence gap (Imran et al., 2023). Responses to metal-containing engineered nanomaterials may likewise be cultivar- and dose-dependent; however, such findings should be distinguished from evidence specifically addressing MNP–metal(loid) co-exposure. ZnO nanoparticles and PE MPs altered seedling growth, physiology, and Zn accumulation differently between two rice cultivars (Tan et al., 2022). These endpoint-level responses do not by themselves identify the processes controlling metal(loid)s transfer to edible tissues. The following section therefore examines how MNPs modify soil metal(loid)s speciation, rhizosphere processes, plant uptake and transport, and microbial functions that may jointly determine these outcomes.

Studies should be interpreted according to exposure system and endpoint. Hydroponic and seedling experiments provide mechanistic indications of root uptake and physiological stress, whereas soil pot experiments and, particularly, field studies with mature grain are required to support conclusions about rice food-safety risk. Grain concentration measurements should further be accompanied by chemical speciation and, where feasible, bioaccessibility assessment. **Table 1**.

**Table 1:** Summary of reported effects, underlying mechanisms, and key influencing factors of co-exposure to micro-/nanoplastics and metal(loid)s in rice systems.

Metal(loid)	Polymer/form	Size	Dose–timing	Hydroponic /soil	Rice stage	Grain	Endpoints	Co-exposure outcome	Mechanism	Ref
Cd	PS-MPs	100 ± 4.47 nm	PS-MPs: 10/20/40 mg L^−1^; Cd: 0.05 mg L^−1^; 3 weeks	Hydroponic	seedling roots	No	root length; fresh/dry weight; root Cd; O2•−; MDA; protein carbonylation; SOD; POD; HSP70; root necrosis	low PS (10–20) partly alleviated Cd; PS = 40 increased Cd accumulation & oxidative injury (dose-dependent; non-additive)	low-dose: ↓Cd bioavailability & ↑antioxidant defenses; high-dose: ↑Cd accumulation & root homeostasis disruption	(Liu et al., 2022)
Cd	PE-MPs	~148 µm	mPE 0.5%/1% (w/w) + Cd 1.5/5 mg/kg; flooded pot; harvest ~maturity (~180 d)	Flooded paddy soil	mature period	Yes	growth (height/biomass); tissue Cd (roots/stems/leaves/seeds; BCF/TF); soil pH & MC; Cd fractions (DTPA/total); leaf SOD/POD/CAT & MDA; soil enzymes & microbial diversity	mPE+Cd reduced growth (~4.1–13.8%) & increased tissue Cd, esp. 5 mg/kg Cd + 1% mPE; microbial diversity ↓; available Cd pools shifted	mPE alters soil condition (pH/MC) & microbial processes → ↑Cd bioavailability/transfer + oxidative stress amplification (risk amplification; non-additive)	(Gao et al., 2026)
Cd	PET MPs; PVC MPs (PET+PVC 0.5%+0.5%)	20–500 µm	PET 1% or PVC 1% (w/w); palygorskite-bound Cd 0.3 mg/kg; 21 d seedlings → transplant; harvest ~120 d	Flooded paddy soil	mature period	Yes	brown-rice Cd; rhizosphere pH; root oxidative injury (MDA, POD); multi-omics (microbiome + pathways)	PET: ↑Cd remobilization (+132.43%), rhizosphere pH 7.05→6.30, brown-rice Cd 0.03→0.06 mg/kg. PVC: no pH acidification but ↑root oxidative stress & ↑Cd uptake	PETase-carrying Acidobacteriota enrichment + suppressed OsNRT2.3 → rhizosphere acidification → mineral-bound Cd release; PVC: barrier damage → enhanced Cd uptake	(Zhou et al., 2026)
Cd	MPs (polymer not specified)	0.5–5 mm (SEM)	Cd in soil 1.5 mg/kg; soil aged 30 d; MPs 0.1%/0.3%/1% added after 5 d pre-incubation; flooded water level 3 cm; 28 d; sampling d3/7/14/28	Flooded paddy soil (incubation)	NR	No	soil pH & Eh; DOC (incl. 3D-EEM); Cd speciation (Fe–Mn bound Cd, available Cd; Tessier+ICP-MS); Fe speciation (HCl-extractable Fe(II)); S species & reduced S (AVS/LMS/MS); 16S rDNA/SRB community; MP surface elements (SEM-EDS: C,N,S,Fe,Cd)	MPs increased Cd availability/release risk (speciation/available Cd); discussed as Eh-dependent; also altered Fe/S dynamics & microbial community	MPs-released DOC drives Fe(III) reduction → promotes Cd release; MPs also inhibit sulfate reduction → reduces CdS immobilization; overall speciation mainly Eh-dependent	(Wang et al., 2024)
Cd	PS-MPs	1–10 µm (S-PS); 50–150 µm (B-PS)	Cd 2.5 mg L^−1^; PS 50 or 500 mg L^−1^; 14 d; PS-MPs ± Cd	Hydroponic	seedling	No	total height; shoot/root height; root length; biomass; photosynthetic pigments (Car, Chl a/b); POD/SOD/CAT; MDA; Cd uptake & TF; nutrient elements (micro: Mn,Zn,Fe,Cu,B,Mo; macro: S,P,K,Mg,Ca)	PS generally exacerbated Cd toxicity; interaction type size & concentration dependent (reported synergy/antagonism in indices)	promotes Cd transfer/accumulation and nutrient imbalance; also obstructs root openings/water-nutrient uptake → oxidative stress	(Jiang M. et al., 2024)
Cd	PS-NPs	50 nm	NPs: 10 & 50 mg L^−1^; Cd: 20 µM; 2-week seedlings	Hydroponic	seedling	No	growth; chlorophyll; Cd subcellular distribution (CW vs protoplast; CW-bound Cd↑ to 76.8% at PS10-Cd); CW integrity (TEM/AFM); Cd translocation to shoots (%; 34.4% at PS50-Cd); oxidative stress (H2O2,O2•−,MDA) & antioxidant enzymes (SOD,CAT,POD)	“double-edged sword”: low-dose NPs mitigated Cd phytotoxicity; high-dose aggravated	low PS10-Cd: Cd immobilized in CW; CW integrity reinforced (carbohydrate & lignin-linked pathways) → restrict entry. High PS50-Cd: ↑CW permeability → ↑Cd translocation; ROS burst + impaired CW biosynthesis	(Xu et al., 2025)
Cd	PP-MPs	6.5 & 13 µm	PP 100 mg L^-1^ + Cd 5.0 mg L^−1^ (Cd^2+^) during germination (D3/D7)	Aqueous (Petri dish/filter paper)	germination & early seedling	No	germination potential/rate; GI (D3,D7); vigor index/MGT; root & stem growth; CAT/POD/SOD	size-dependent: 6.5 µm PP+Cd synergistic (worse); 13 µm PP+Cd antagonistic (alleviated)	hydrophobic MP–Cd interaction; size-dependent phytotoxicity; Cd-induced oxidative stress (antioxidant enzymes inhibited)	(Kaur et al., 2022)
Cd	PVC-MPs under acid-rain conditions	avg. 75 µm	Cd 0.3/3/10 mg L^−1^; MP 10 g/L; AR pH 4.0; start at 21-day seedlings; 7 d exposure	Hydroponic (0.5× Kimura B)	seedling	No	growth (height,dry wt,root:shoot), chlorophyll; ROS (NBT/H2O2/MDA); antioxidant (SOD); cell damage (Evan’s blue); Cd accumulation; transcriptome/qPCR (OsNramp5, OsHMA2, OsABCG36; MAPK/glutathione; GSTs; metallothioneins)	low Cd: MP+AR no significant effect; high Cd (10 mg L^-1^): alleviated Cd stress (↓ROS/↓MDA; ↑SOD; ↓cell death; ↓Cd accumulation)	high-Cd antagonism: suppress Cd transport gene expression → lower Cd; enhance antioxidant/glutathione responses → reduce oxidative stress	(Xie et al., 2024)
Cd	polyethylene MPs	10 µm (diam.)	CdCl2–200 mg kg^−1^ soil (stabilized 2 months); MPs 1% (w/w) added; BC 2% (w/w) 1 week later; MT 100 µM foliar spray 20 d post-transplant	Soil pots (water depth 2–3 cm)	seedling→maturity (90 d)	Yes	biomass & grain yield/1000-grain wt; Chl-a/b/Cart.; RWC; MDA & H2O2; antioxidant enzymes; proline; Cd accumulation	BC+MT strongly alleviated MP-Cd stress (growth/chlorophyll restored; oxidative stress ↓; Cd accumulation ↓)	BC adsorption/immobilization ↓Cd bioavailability; MT ↑antioxidant/biosynthesis genes & ↓Cd transporter genes	(Yang et al., 2026)
Cd	PVC MPs + NZMB	50 µm	CdCl2–20 mg kg^−1^ soil (stabilized 2 months, dark, 70% FC) + MPs 1% (stabilized 1 week) + NZMB 2% (stabilized 1 week); grown in pots (May–Jul 2024)	Soil pot	seedling→maturity	Yes	biomass/yield & traits (root length/biomass, plant height, tillers, 1000-grain wt, grain yield); chlorophyll; leaf RWC; soil nutrients; Cd in tissues; oxidative markers/antioxidant/osmolytes; gene expression	NZMB strongly alleviated Cd+MP toxicity (yield & chlorophyll restored; oxidative stress ↓; Cd toxicity ↓)	↑soil properties (pH/OC/nutrient availability) + ↓Cd availability/accumulation; activated stress-response & energy-metabolism genes; strengthened antioxidant/osmolyte responses	(Jinhua et al., 2026)
As	PSMP + PTFE (co-exposure with As)	PSMP/PTFE: S = 0.1–1 µm; L = 10–100 µm	As soil 1.4/24.7/86.3 mg kg^−1^; MPs 0.25% or 0.5% (w/w; 0/5/10 g per 2 kg soil); pot (3 seedlings/pot); harvest yield (grain)	Soil pots	NR (to grain harvest)	Yes	root/stem/leaf bio-mass; grain yield (%); root activity; Fe–Mn plaque Fe/Mn; As uptake & speciation (As(III), As(V), MMA, DMA); hemoglobin content/structure; starch synthase (SSS)/pyrophosphorylase (PS) in grains; AGP/SSS; ROS “oxidation burst”	at As 86.3: 0.5% S-PSMP ↓grain yield 30.7%; 0.5% S-PTFE ↓~19.4%; PSMP/PTFE reduced As uptake and grain yield overall	MPs aggregated near roots; inhibit root activity & reduce Fe–Mn plaque formation; PSMP/PTFE disrupt Hb tertiary structure (↓Hb) while As(III) ↑Hb via ROS; oxidation bursts ↓AGP/SSS & starch accumulation	(Dong et al., 2022)
As	PSMP + PTFE-MP	PSMP 0.1–1 µm (S1); PTFE 10–100 µm (S2)	As-contaminated soil 1.4/24.7/86.3 mg kg^−1^; MPs 0.25% (5 g/2 kg) or 0.5% (10 g/2 kg); added at start; rhizosphere harvested at harvest	Soil pots	tillering/booting→maturity; rhizosphere sampling	No	soil pH; As(III)/As(V); urease/acid phosphatase/protease/dehydrogenase/peroxidase; AN/AP; SOM; microbial abundance (↓Proteobacteria; ↑Chloroflexi/Acidobacteria)	PSMP and PTFE with As ↓soil pH and ↓As bioavailability	MPs reduce As(III)/As(V) and disrupt enzyme structure/function; shift rhizosphere microbes & reduce N/P availability; small particles stronger	(Dong et al., 2021)
As	PE MPs; PLA MPs	mean ~100 µm	As: 30 & 90 mg kg^−1^ soil; MPs 0.25% (w/w; mixed with 5 kg soil/pots); greenhouse pot 60 d	Soil pots	vegetative (harvest ~60 d; no maturity stated)	No	shoot/root biomass & length; As in roots/shoots; iron plaque (DCB-Fe); As fractions (F1–F6; bioavailable vs residual); Chl a/b/total; oxidative stress (POD/SOD); O2•−	low As (30): antagonistic (MPs alleviate growth inhibition; ↓As in roots/shoots); high As (90): synergistic esp. PE (↑As uptake & worsened oxidative stress)	MPs compete for As adsorption and alter bioavailability + iron plaque; As30 shifts toward less-bioavailable As & higher plaque; As90 promotes more bioavailable As and reduces plaque → stronger oxidative stress; PE > PLA	(Wu et al., 2025b)
As	PE MPs; PLA MPs	~100 µm	NaAsO2 30 & 90 mg kg^−1^; MPs PE or PLA 0.25% (w/w); paddy pot to harvest; vegetative + grain filling	Soil pots	vegetative + spike/grain filling	Yes	biomass (2A roots/stems/leaves); spike/grain filling; As in roots/cell wall/soluble/organelles; As fractions & bioavailable proportion; Fe plaque/iron plaque; oxidative stress (O2•−, MDA); SOD/CAT/POD; genotype Yakahang 2A vs Yahui 2119	PE worsens (growth ↓; oxidative stress ↑; As accumulation ↑); PLA mitigates (growth ↑ in some traits; As toxicity ↓); genotype-specific (PE stronger in high-As genotype)	PE increases bioavailable As fractions (↑F1–F3), more As uptake & oxidative stress; PLA shifts fractions toward non-bioavailable (↑F5–F6), reduces As entry; MPs alter iron plaque	(Wu et al., 2025a)
Pb	PVC MPs + BC + MT	10 µm	Pb (NO3)2–250 mg kg^−1^ soil; MPs 1% (w/w) + BC 2% (w/w); MT 100 µM foliar spray at 20 d post-transplant (3 d); soil stabilized 2 months; MPs equilibrated 2 weeks	Soil pots; irrigation depth 2–3 cm	transplant (~25 d)→maturity	Yes	biomass & grain yield; harvest index; oxidative stress; chlorophyll/photosynthetic traits; hormones (IAA, GA, ABA); proline/osmolytes; soil nutrients (TN/AP/AK) & pH; Pb availability/uptake; grain Pb; gene expression	Pb+MPs decreased biomass & grain yield (−25.26%, −41.51%); BC+MT alleviated and increased (+18.20%, +39.59%)	BC+MT increased pH/nutrient availability; enhanced antioxidant & osmolytes; modulated hormones; suppressed Pb uptake genes (e.g., OsHMA9, OsNRAMP5) → ↓Pb accumulation	(Du et al., 2026)
Cu^2+^	PS microplastics	300 mesh	PS 50 mg L^−1^; Cu 20 mg L^−1^; PS+Cu; measured day 7	Hydroponic (culture solution)	seedlings (1-week; root fully immersed)	No	root length; stem length; fresh/dry weight; POD (roots & shoots); Cu content	combined: no significant additional synergistic/additive toxicity; PS+Cu mitigated Cu (↓Cu accumulation; root ~ CK)	PS adsorbs Cu^2+^ → ↓Cu bioavailability/uptake; PS also suppressed antioxidant response (↓POD)	(Jin et al., 2024)
Zn	PS microplastics (PS-NPs)	~80 nm (nominal)	PS 10 mg L^-1^; ZnSO_4_ 1.2 μM; start when seedlings had 3 fully developed leaves; 21 d; sampled after 21 d	Hydroponic Hoagland	seedlings (21 d; roots/stems/leaves sampled)	No	growth (root/stem length); photosynthesis (Pn, Ci, Tr, Gs); ROS (H2O2, MDA); SOD/POD/CAT	PS inhibited growth/photosynthesis and ↑ROS; Zn significantly alleviated (↓MDA/H2O2; ↑SOD/POD/CAT; recovery in root/stem)	organ-specific: shoots—optimizes nitrogen metabolism to restore photosynthesis; roots—regulates carbon metabolism to reduce ROS; also ↑zinc transporter genes (OsZIP4/6/7/8/9/10)	(Liu et al., 2025)
Hg	PLA-MPs	50 µm	PLA 0.2% & 0.5% (soil wt); THg via HgCl2 adjusted to 3200 μg kg^−1^; flooded 7 d before planting; pot 180 d (Jun20–Oct12 2024); drained at maturity (Sep28)	Soil (flooded paddy pot)	maturity (grain harvested)	Yes	THg/MeHg in soil & rice organs (roots/leaves/grains); grain Hg; soil properties (incl. pH); 16S rRNA diversity; hgcA/hgcB; methylation taxa (Desulfobacterota)	PLA-MPs enhanced MeHg production; grain THg (Hgrr) ~130.46 μg kg^−1^ (~6-fold CK); α-diversity ↓ but community composition ~unchanged	PLA-MPs upregulated hgcA/hgcB; affected soil pH (negatively related to hgcA); Desulfobacterota dominant methylation taxon; hgcA/hgcB positively correlated with grain Hg	(Wenwen et al., 2025)
Pb, Cd, and other heavy metals	MNPs (review synthesis)	NR	NR	fragrant-rice/paddy systems	NR	Yes	2-Acetyl-1-pyrroline (2-AP) biosynthesis/contents; HM/MNP root vs grain accumulation pattern	review-level: HMs & MNPs threaten fragrant rice safety; root accumulation complicates assessment of grain quality traits; context-dependent mixture effects	root accumulation + disrupted metabolism; 2-AP biosynthesis should be monitored under increasing contaminants; nutrient/phytohormone strategies suggested	(Imran et al., 2023)

The direction of co-exposure effects should not be interpreted as synergistic or antagonistic unless an explicit interaction model and appropriate single-stressor controls were applied.

MP, microplastic; MNP, micro- and nanoplastic; NP, nanoplastic; metal(loid)s, metals and metalloids; Cd, cadmium; As, arsenic; Pb, lead; Cu, copper; Zn, zinc; Hg, mercury. PS, polystyrene; PE, polyethylene; PET, polyethylene terephthalate; PVC, polyvinyl chloride; PP, polypropylene; PTFE, polytetrafluoroethylene; PLA, polylactic acid. ROS, reactive oxygen species; MDA, malondialdehyde; SOD, superoxide dismutase; POD, peroxidase; CAT, catalase; HSP70, heat shock protein 70. DTPA, diethylenetriaminepentaacetic acid; DOC, dissolved organic carbon; Eh, redox potential; MC, moisture content; BCF, bioconcentration factor; TF, translocation factor; DCB-Fe, dithionite-citrate-bicarbonate extractable iron (iron plaque proxy); AN/AP, alkaline phosphatase/acid phosphatase; AVS, acid volatile sulfide; LMS, labile monosulfide; MS, metal sulfide; SRB, sulfate-reducing bacteria; 16S rDNA, 16S rRNA gene. H2O2, hydrogen peroxide; O2•−, superoxide anion. Cu^2+^, copper ion; Cd^2+^, cadmium ion; THg, total mercury; MeHg, methylmercury; hgcA/hgcB, mercury-methylation genes; 2-AP, 2-acetyl-1-pyrroline; AGP, ADP-glucose pyrophosphorylase; SSS, starch synthase; PPase, pyrophosphorylase. TEM, transmission electron microscopy; AFM, atomic force microscopy; SEM-EDS, scanning electron microscopy with energy-dispersive X-ray spectroscopy; 3D-EEM, three-dimensional excitation–emission matrix fluorescence; qPCR, quantitative PCR; w/w, weight/weight. ↓: indicates a decrease/reduction; ↑: indicates an increase/elevation; →: indicates a cause leading to / resulting in / shifting toward a particular outcome

## 4 Mechanisms and context-dependent outcomes of MNP–metal(loid) co-exposure in rice: integrating metal(loid) availability, plant responses, and tissue partitioning

An evidence hierarchy is applied to mechanistic claims: causal wording is reserved for direct measurements (and experimentally separated intermediates), hypothetical wording is used for inference from cross-study patterns, and transcriptomic/omics results are presented as correlative, hypothesis-generating associations. The mechanistic logic is organized along the continuum from soil processes to mature-grain endpoints, where MNP-driven changes in metal(loid) speciation/availability may propagate into rhizosphere conditions, root transport regulation, and ultimately determine mature-grain loading.

### 4.1 Soil: MNPs reprogram metal(loid)s availability and speciation

Availability and Speciation

In flooded–aerated redox-cycling paddy soils, metal(loid)s behavior is driven mainly by transformation chains—mineral adsorption/desorption, shifts among exchangeable pools, and conversion to organically complexed forms—rather than being determined solely by total metal(loid) concentrations (Li et al., 2025; Yu et al., 2026). As co-contaminants, MNPs can modify soil physicochemical processes and microbial functions through polymer-specific surface chemistry and degradation signatures (e.g., functional groups and aging-dependent surface states for PET, PVC, PLA, PE), size- and concentration-dependent changes in diffusion and interfacial contact frequency, and the plastisphere microenvironment (Lin et al., 2022; Wang et al., 2024; Zhu et al., 2024; Zhou et al., 2026). Depending on polymer properties and soil redox conditions, MNPs may redistribute metal(loid)s among solid-phase and dissolved pools, and the apparent direction of metal(loid) availability to rice is thus variable. Consequently, co-exposure commonly yields non-equivalent outcomes, with departures from additive expectations (Wang et al., 2024; Xu et al., 2025; Zhou et al., 2026).

Evidence for Cd is strongly shaped by polymer-specific speciation rearrangements. PET and PVC both weaken mineral stabilization of Cd (e.g., on palygorskite), but they differ in the key de-stabilization steps and the associated bioavailability outputs. Under PET, rhizosphere/local interfacial acidification has been observed in association with nitrogen-metabolism regulation: rhizosphere pH was reported to decrease from 7.05 to 6.30 with inhibition of OsNRT2.3, which is consistent with Cd remobilization from mineral-fixed pools (mineral-fixed Cd remobilization: F1 = +132.43%) and with the co-occurrence of increased Cd in brown rice (from 0.03 to 0.06 mg/kg). By contrast, for PVC, acidification-related evidence appears less prominent; instead, root-interface oxidative-stress responses and impairment of the interfacial barrier have been associated with Cd destabilization via altered effective ion flux and ion-exchange efficiency, resulting in an endpoint trajectory that differed from PET (Zhou et al., 2026). Overall, different polymers may be associated with different “steps” along the transformation chain; however, stepwise causal dominance among intermediate processes requires direct experimental separation within the same study design.

For As, reprogrammed availability is similarly controlled by MPs/NP type, with strong contributions from particle-size and concentration thresholds to how “speciation inputs” are partitioned. While irrigation water supplies background As, porewater As is mainly regulated by MPs characteristics: high MPs concentration (3% w/w) increases porewater As by 2.8–4.5 times versus low (1% w/w), and PLA releases more As than PE by ~1.9–2.3 times (Yu et al., 2026). This pattern is consistent with a shift toward exchangeable/mobile chemical pools that are more readily captured by rhizosphere exchange processes, potentially increasing uptake flux. However, smaller particles do not necessarily yield a linear increase; nano-scale interfacial effects can redirect (and even reverse) the dominant speciation transformation trajectory, contributing to departures from additive expectations. Plant-side feedback supports chain-level risk amplification: under co-exposure, As in shoots increases by 70.9% and in roots by 24.5% (vs. As-only), with a 16.0% decrease in abscisic acid (ABA), 22.5% suppression of iron plaque formation, oxidative (+7.0%) and transport (+37.0%) enhancement, and 2.1–2.7-fold upregulation of aquaporin/water-channel–related genes (Jiang Y. et al., 2024). Thus, compounded As risk likely reflects both altered soil availability and MPs-driven feedback that moves rhizosphere/physiology into a more bioavailable speciation interval.

For Pb, current evidence more often points to availability changes inferred through endpoint recovery or steady-state rebuilding rather than direct speciation data. For example, biochar plus melatonin (BC+MT) reverses negative deviations in biomass (BY) and grain yield (GY) under Pb+MPs (BY + 18.20%, GY + 39.59%), suggesting that co-toxicity outputs can be recalibrated by matrix stabilization and detoxification-related regulation; this should not be equated with Cd/As-specific transformation pathways without direct morphological/speciation quantification (Du et al., 2026). For Hg, evidence emphasizes MPs-driven preferential reprogramming toward highly toxic forms: methylmercury formation depends on microbial methylation, and MPs may enhance Hg flow through the transformation chain by altering soil DOM composition, local redox microenvironments, and functional taxa. Under PLA-related co-exposure, grain Hg can reach 130.46 μg/kg (~sixfold of control) with upregulated hgcA/hgcB (methylation key genes). hgcA is negatively correlated with pH; dominant methylation-associated taxa include Desulfobacterota, and hgcA/hgcB expression correlates positively with grain Hg enrichment (Wenwen et al., 2025). Overall, Hg risk amplification that departs from additive expectations may arise because microbial functional networks redirect.

### 4.2 Rhizosphere: MNPs drive iron/sulfur/ligand-coupled metal(loid)s transformation

In the rice rhizosphere, metal(loid)s speciation is co-governed by Fe cycling (Fe(III)/Fe(II)), sulfur transformations (sulfate reduction and reduced sulfur formation), and DOM-mediated ligand complexation (Wang et al., 2024). Compared with bulk soil, rhizosphere conditions are more dynamic due to continuous root exudation (proton/ion transport and redox regulation), microbial enrichment near roots/particles, and plastisphere formation. Following MNP entry, polymer-specific interfacial niches can reconfigure Fe/S/ligand boundary conditions, which may shift transformation patterns away from mineral-stabilization routes toward trajectories that are closer to exchangeable forms and/or plant-transport-captured pools. Such reconfiguration is consistent with the frequent appearance of non-equivalent outcomes showing departures from additive expectations (Xu, 2026; Zhou et al., 2026), although stepwise causal dominance among intermediate processes requires direct experimental separation.

For Cd, PET and PVC appear to drive Cd reprogramming through non-identical rhizosphere nodes. Under PET, acidification-associated microbial enrichment was reported to lower rhizosphere pH from 7.05 to 6.30 and suppress OsNRT2.3 expression, with these changes associated with Cd remobilization from mineral-bound pools. This pattern was reflected in enhanced remobilization from palygorskite (F1, + 132.43%) and increased brown-rice Cd (0.03 to 0.06 mg kg^-1^). Under PVC, the dominant evidence points to oxidative root-interface stress: oxidative indicators increased (MDA + 78%, POD + 60%), and Cd destabilization is more likely mediated by impaired root-surface barrier integrity and altered ion selectivity, resulting in a trajectory that differed from PET (Zhou et al., 2026). Plastisphere coupling further reshapes Fe/S–Cd fate: plastics can enrich taxa linked to Fe(III) reduction and sulfate reduction under flooded conditions, increase Fe(II) and sulfur availability, and convert background rhizosphere Fe–S activity into localized plastic-surface reactions (Wang et al., 2024; Zhu et al., 2024). Sequential extraction showed that MPs increased available Cd and decreased Fe–Mn oxide-bound Cd under Cd–MP co-contamination, with effects particularly pronounced at days 7 and 14 (Wang et al., 2024). Mechanistically, MPs may enhance DOM-associated iron–organic ligand formation and accelerate reductive dissolution of iron minerals while decreasing amorphous iron availability; meanwhile, MPs can increase sulfate-reducing bacteria (e.g., Streptomyces, Desulfovibrio) and reduced sulfur species, potentially increasing local Fe–S–Cd co-precipitation/re-stabilization potential. Whether rhizosphere risk ultimately increases or decreases may depend on exposure duration and on how root-driven dynamics re-disturb interfacial stability. Overall, Cd reprogramming can be interpreted as a plastic-niche shift in Fe/S/ligand coupling that reshapes temporal dynamics and spatial distribution (Wang et al., 2024).

For As, NP effects are more consistent with destabilization of an “iron plaque–phytohormone–transport” coupling at the root interface. Under As(III) + NPs exposure, NPs did not markedly affect rice growth alone but amplified As phytotoxicity by 9.4–22.8%, increased As accumulation by 24.5% in roots and 70.9% in shoots, decreased ABA by 16.0%, upregulated aquaporin-related genes by 2.1–2.7-fold, and inhibited root iron-plaque formation by 22.5%. The inhibition of root-surface iron-plaque formation by 22.5% may weaken root-surface As retention. NPs also disrupted SA-, JA-, and GSH-related metabolism, with higher in planta As oxidation by 7.0% and translocation by 37.0% while reducing detoxification capacity (Jiang Y. et al., 2024). Separately, aged nano-sized PLA was shown to co-transport with As through the xylem and accumulate in leaf veins, indicating that plastic type and size can alter tissue-scale As partitioning (An et al., 2025a).

For Hg, evidence points to DOM–Fe–S coupling as a shared driver, although direction may differ from Cd/As. For example, under PVC–MPs, potentially available MeHg can decrease across rice–soil contexts. This pattern may reflect PVC–MPs shifting DOM composition/complexation capacity toward Hg^2+^, while reshaping sulfate and dissolved iron supply conditions that affect methylation efficiency and the structure/expression of methylation-related microbial communities (including hgcA-associated pathways). Overall, regardless of whether MeHg flux increases or decreases, MPs can reconfigure DOM ligand environments and Fe/S supply, shifting Hg speciation-generation toward different MeHg flux states (Yang et al., 2022).

### 4.3 Plant response: barrier integrity, metal(loid)s entry flux, and tissue allocation under co-exposure

Under metal(loid)–plastic co-exposure, rice toxicity is modulated by both metal(loid) entry across root barriers and downstream tissue allocation. Root-barrier function involves cellular redox status and cell-wall integrity, as well as root-surface retention mechanisms such as iron plaque. Plastic-induced perturbations may shift metal(loid)s uptake, root-to-shoot transport, and tissue accumulation, thereby contributing to mixture-specific and potentially non-additive outcomes (Xu C. et al., 2023; Jiang Y. et al., 2024; Xu et al., 2025). Two mechanistic routes are commonly proposed for this propagation: (i) effects on root-barrier integrity that influence metal(loid) entry and root-to-shoot translocation, and (ii) changes in stress- and transport-related gene regulation. In Cd-exposed rice, low NP doses were reported to enhance root cell-wall thickening and Cd retention, whereas high doses were associated with oxidative stress, increased cell-wall permeability, and higher Cd uptake and shoot translocation. Consistently, low and high NP concentrations elicited opposing antioxidant and transcriptomic responses in roots under Cd co-exposure (Xu et al., 2025; Liu et al., 2026).

For Cd co-exposure, root responses to NPs were concentration dependent. At 100 mg L^−1^ NPs plus 0.5 mg L^−1^ Cd, root length, root-tip number, and root surface area were 14%, 22%, and 4% lower than under Cd alone, respectively, suggesting synergistic phytotoxicity. In contrast, 10 mg L^−1^ NPs reduced MDA and increased SOD/POD activities, whereas 100 mg L^−1^ NPs were associated with higher lipid peroxidation and decreased antioxidant-enzyme activities. Transcriptomic and WGCNA analyses further linked the high-NP/Cd response with elevated expression of OsGRP162 and OsCYP2, and with a shift from glutathione-metabolism enrichment at low NP exposure toward MAPK-signaling enrichment at high NP exposure (Liu et al., 2026). PET exposure was associated with increased brown-rice Cd concentrations from 0.03 to 0.06 mg/kg, linked to rhizosphere acidification and remobilization of mineral-associated Cd (Zhou et al., 2026). In a separate rice study, PP microplastic–Cd co-exposure increased Cd concentrations across roots, stems, leaves, and grains, with particularly pronounced increases in stems and grains relative to Cd exposure alone, indicating that plastic type can modify both Cd uptake and tissue-level accumulation patterns (Kaur et al., 2022).

For As, amplification is mainly reflected in destabilized coupling among the root-surface barrier (iron plaque), hormone regulation, and cross-interface transport. Under As(III) + NPs exposure, growth effects may not always change markedly, yet toxicity and accumulation were reported to increase: toxic effects rose 9.4–22.8%, while As accumulation increased by 24.5% in roots and 70.9% in shoots versus As-only. NPs decreased ABA by 16.0% and upregulated water-channel genes by 2.1–2.7-fold, which may increase the physiological feasibility of As entry and internal movement. In parallel, NPs inhibited iron-plaque formation by 22.5%, which could weaken root-surface retention for As and thereby contribute to higher As accumulation in root tissue. NPs also perturbed SA/JA- and GSH-related metabolism, with increased oxidative (+7.0%) and transport (+37.0%) processes alongside reduced detoxification buffering, consistent with higher flux transmission from root surface to vascular tissues (Jiang Y. et al., 2024). Reduced iron plaque does not always translate to increased As uptake. In pot-grown indica rice, PS and PTFE MPs decreased root activity, iron plaque, and As uptake, accompanied by shifts in Geobacteria and Anaeromyxobacter (Dong et al., 2022). Meanwhile, MPs and As inhibited starch-synthesis enzymes and reduced grain yield, suggesting that As accumulation and productivity may respond differently across exposure conditions. At the rhizosphere–soil level, PS and PTFE MPs also decreased soil As(V) and As(III), available N and P, microbial abundance, and several enzyme activities under As exposure (Dong et al., 2021). Together, these results indicate that reduced As bioavailability may coexist with compromised rhizosphere nutrient cycling and microbial functioning.

For Pb, Pb+MP co-exposure reduced rice biomass and grain yields by 25.26% and 41.51%, respectively. Combined BC+MT application partly alleviated these losses by increasing biomass and grain yields by 18.20% and 39.59%, respectively, while enhancing antioxidant activity, improving IAA/GA-related hormonal status, reducing Pb availability, and downregulating Pb-uptake genes (Du et al., 2026). Under PLA-MP exposure, mean grain Hg concentration reached 130.46 μg kg^-1^, approximately sixfold that of the control. This increase was accompanied by enhanced soil MeHg formation and upregulation of Hg-methylation genes hgcA and hgcB, whose expression was positively correlated with grain Hg accumulation (Wenwen et al., 2025). Across metal(loid)s–plastic systems, evidence to date suggests that polymer identity and particle dose, together with metal(loid)-specific soil–root processes,

### 4.4 Molecular mechanisms: network rewiring of detoxification, metal(loid)s transport, and stress signaling

Physiological and transcriptomic (including multi-omics) evidence indicates that metal(loid)s–plastic co-exposure alters antioxidant capacity, glutathione-related metabolism, stress signaling, and ion-transport–associated responses in rice. Response direction and magnitude depend on polymer identity and co-exposure context, reflected in polymer-specific rhizosphere processes and in non-additive transcriptional patterns reported for specific PVC-containing mixtures. Across these studies, altered root entry conditions and shifts in tissue-level transport regulation are consistently discussed as contributors to differences in metal(loid)s accumulation endpoints (Jiang Y. et al., 2024; An et al., 2025a; Xie et al., 2026; Zhou et al., 2026), although the relative causal weight of each step can vary among experimental designs.

For Cd, plastic-associated effects can involve both rhizosphere Cd destabilization and dose-dependent changes in root defensive physiology. PET- and PVC-associated pathways differ: PET has been linked mainly to rhizosphere acidification and remobilization of mineral-associated Cd, whereas PVC is more strongly associated with oxidative-stress responses (Zhou et al., 2026). In rice roots, Cd–NP co-exposure also coincides with altered root morphology and coordinated activation of stress-responsive and transport-/defense–linked transcriptional modules, including OsGRP162 and OsCYP2, together with root-development–related responses (Liu et al., 2026). In addition, low NP exposure has been associated with maintenance of antioxidant defenses and improved Cd retention at the root cell-wall level, whereas high NP exposure corresponds to aggravated oxidative stress, impaired cell-wall integrity, and enhanced Cd movement toward shoots (Xu et al., 2025). Taken together, the Cd-related molecular responses show a dose-dependent shift from compensatory defense toward compromised root retention and transport regulation.

For As(III)+NP, reported physiological readouts include ABA downregulation, aquaporin-related gene upregulation, reduced root iron-plaque formation, and disruption of SA/JA/GSH metabolism, alongside increased in planta As oxidation and translocation with reduced detoxification capacity (Jiang Y. et al., 2024). Across rice–As–plastic systems, mixture effects are not uniform and vary with As concentration, polymer identity, particle size, and plant compartment: PE and PLA effects shift from antagonism at low As to synergistic toxicity at high As, while nano-PLA enhances As uptake whereas larger PLA particles inhibit it (An et al., 2025b; Wu et al., 2025b).

For Pb+MP, growth inhibition is accompanied by oxidative stress and disturbed hormonal regulation. BC+MT has been reported to partly restore antioxidant and hormonal homeostasis, reduce soil Pb availability, downregulate Pb-uptake–related genes, and improve biomass and grain yield outcomes (Du et al., 2026). For Hg, increased MeHg formation coincides with substantially higher Hg accumulation in rice grains, supporting the view that PLA-MP-associated changes in soil Hg speciation can strongly shape the grain-level food-safety endpoint (Wenwen et al., 2025).

Overall, available evidence supports a network-rewiring perspective in which metal(loid)–stress responses are redistributed across dominant detoxification/antioxidant nodes, hormone–transport modules, rhizoplane plaque functions, and toxic-species generation pathways. Because polymers and particle scales selectively reshape the activity and coupling of these modules, co-exposure yields non-additive, context-dependent endpoint signatures across growth, yield, and edible-organ element accumulation.

### 4.5 Microbial mechanisms: MNPs reshape community assembly and functional metal(loid)s cycling

MNPs can create plastisphere microenvironments that differ from bulk soil in microbial composition and metal(loid)-cycling functions. In rice systems, biodegradable polymers can differentially alter microbial niche breadth and deterministic versus stochastic assembly processes, with consequences for Cd accumulation (Lin et al., 2022). PET and PVC also induce polymer-specific rhizosphere microbial and metabolic pathways associated with Cd remobilization, while plastisphere-associated Fe and sulfate reduction can increase Cd availability (Zhu et al., 2024; Zhou et al., 2026). Evidence from Hg systems further shows that plastic-associated microbial and geochemical changes may either enhance or reduce MeHg production depending on polymer and soil context (Yang et al., 2022; Hao et al., 2023). Beyond metal(loid)s cycling, MPs can alter broader paddy-soil functions: PLA, LDPE, and PP shifted microbial communities, DOM characteristics, and greenhouse-gas emissions, with ecosystem multifunctionality varying by polymer type and dose (Zhang et al., 2024).

Cd evidence indicates that Fe-coupled Cd mobilization pathways can be polymer-specific. PET and PVC can both destabilize palygorskite-immobilized Cd, but through distinct rhizosphere routes. PET induced stronger remobilization of mineral-fixed Cd (F1: +132.43%), accompanied by decreased rhizosphere pH (7.05 to 6.30) and increased brown-rice Cd concentration (0.03 to 0.06 mg kg^-1^). PET also suppressed OsNRT2.3 and enriched the keystone taxon Gaiella through homogeneous selection. In contrast, PVC did not acidify the rhizosphere but was characterized by severe root oxidative stress (MDA + 78%; POD + 60%) and distinct microbial metabolic profiles, including redirection of carbon flux toward the pentose phosphate pathway and increased genetic potential for acetate-dependent methanogenesis (acs +22.67%). Collectively, these findings support polymer-specific restructuring of rhizosphere processes that are consistent with Fe(III)- and redox-related control of Cd remobilization and bioavailability (Zhou et al., 2026). Environmental context can further modulate whether microbial-mediated changes translate into plant Cd outcomes. In rice seedlings, PVC MPs plus simulated acid rain (pH 4.0) partly alleviated toxicity at 10 mg L^−1^ Cd, reducing root Cd accumulation, cellular damage, ROS, and MDA, while altering Cd transport, MAPK, GST, and metal(loid)s lothionein-related gene expression (Xie et al., 2024). MP effects on Cd toxicity are therefore context- and dose-dependent. Consistent with this context dependence, PET, PLA, and PES MPs generally increased soil Cd availability but decreased Cd accumulation in rice tissues, while altering AMF diversity and community composition. PLA caused the strongest changes in soil properties and plant growth, yet no synergistic interaction with Cd was detected (Liu et al., 2023), indicating that higher soil Cd bioavailability does not necessarily result in greater plant Cd accumulation.

Under flooded conditions, microbial Fe/S-coupled cycling links plastisphere assembly to Cd release risk. MPs increased Cd release risk and decreased Fe–Mn oxide-bound Cd, particularly on days 7 and 14, in association with dissolved organic-substance release, Fe-mineral reductive dissolution, and altered sulfate reduction. MPs also created plastisphere microenvironments whose bacterial communities differed from bulk soil; enrichment of Symbiobacteraceae, Rhodocyclaceae, and Bryobacteraceae was associated with enhanced Fe(III) and sulfate reduction, elevated Fe(II) and S contents, and Cd enrichment at particle interfaces. In rice rhizospheres, Cd stress narrowed bacterial niche breadth, whereas PPC partly alleviated this pressure; PPC-associated abundant and rare taxa were governed predominantly by deterministic and stochastic processes, respectively. Collectively, these findings suggest polymer- and niche-dependent microbial routes of Fe/S-mediated Cd cycling (Lin et al., 2022; Wang et al., 2024; Zhu et al., 2024).

For As and Hg, MNP-driven shifts in microbial metal(loid) transformation pathways can generate polymer- and soil-dependent food-safety risks. In paddy soils, CMPs decreased porewater As by 25–38% but increased %MeAs by 8–23-fold after 98 d, whereas 5% BMPs increased porewater As by 2–9-fold and %MeAs by 11–395-fold; these contrasting outcomes were associated with differences in Fe reduction, As release, substrate availability, and arsM-related microbial groups (Li et al., 2025). For Hg, PLA MPs increased soil hgcA/hgcB expression, which was positively correlated with grain Hg; Desulfobacterota was reported as a major methylating group. PLA MPs enhanced MeHg production and increased grain Hg to 130.46 μg kg^−1^, approximately sixfold above the control (Wenwen et al., 2025). Plastispheres also showed higher MeHg-production potential and distinct methylation-associated communities relative to bulk soil, with Geobacterales and Methanomicrobia linked to methylation potential in yellow and red soils, respectively (Hao et al., 2023). However, PVC MPs reduced labile MeHg in some paddy soils (Yang et al., 2022). Overall, available studies indicate that MNPs reshape microbial community assembly and functional capacities across Fe cycling, sulfur cycling, Hg methylation, and carbon metabolism, while the linkage between microbial changes and grain accumulation is not universally consistent. Microbial transformation shifts can align with grain endpoints in some settings (e.g., PLA-related Hg methylation), yet in other Cd contexts microbial or soil-side changes in bioavailability can decouple from plant tissue loading, indicating that grain accumulation reflects both soil bioavailability and plant partitioning/transport processes rather than microbial alterations alone (Liu et al., 2023; Li et al., 2025; Wenwen et al., 2025).

### 4.6 Context-dependent responses across exposure conditions

Under co-exposure, the direction and magnitude of responses are not determined by overall stress intensity alone, but can shift with particle dose, polymer identity, metal(loid) concentration, and the broader soil–plant–microbial context. For Cd, low NP doses may coincide with partial physiological compensation, whereas higher NP doses are often associated with aggravated injury. For As, both the direction and magnitude of observed outcomes vary with As level and with PLA/PE particle characteristics. For Hg, PLA MPs can enhance methylation-related processes and grain accumulation, while PVC MPs may reduce labile MeHg in some paddy-soil contexts. Overall, these findings indicate a shift in the relative contribution among coupled processes at the soil–root interface, within plant defense networks, and across microbial functional pathways, rather than a single universal response that can be inferred from either stressor alone (Yang et al., 2022; Wu et al., 2025b; Liu et al., 2026).

Because the dominant regulatory processes differ across exposure systems, co-exposure outcomes can vary even among the same metal(loid). In Cd-exposed rice, NP concentration has been reported to accompany a shift from partial compensation toward more pronounced root toxicity: at lower NP doses, oxidative damage markers decreased and antioxidant enzyme activities increased, whereas higher doses increased oxidative injury and impaired root development. For example, at 100 mg L^−1^ NPs plus 0.5 mg L^−1^ Cd, root length, root-tip number, and root surface area declined by 14%, 22%, and 4%, respectively, relative to Cd alone (Liu et al., 2026). Continuous NP exposure has also been linked to a dose-dependent transition in Cd retention, shifting from enhanced immobilization in root cell walls at lower doses to cell-wall disruption and greater shoot translocation at higher doses (Xu et al., 2025). Evidence for As, in contrast, indicates a stronger dependence on organ-specific regulation. Under NP–As(III) co-exposure, transcriptomic responses differed between roots and leaves, with root patterns reported as predominantly additive within the analytical framework of the study, while leaves showed more complex departures (Xu, 2026). In addition, NPs were associated with reduced root iron plaque formation and ABA, increased expression of aquaporin-related genes, and disrupted SA/JA/GSH metabolism, coinciding with higher As accumulation and translocation (Jiang Y. et al., 2024).

In Hg-contaminated systems, a key distinction is the microbial methylation pathway. MNP-associated changes in microbial methylation processes may therefore be particularly relevant to Hg endpoints. In one study, PLA MPs were linked to higher Hg methylation potential, increased hgcA and hgcB expression, and enrichment of methylation-associated taxa such as Desulfobacterota; in the same dataset, total Hg concentration in rice grain reached 130.46 μg kg^−1^, approximately sixfold higher than the control (Wenwen et al., 2025). By contrast, other metal(loid)–MP outcomes may be substantially shaped by the capacity for external restoration of plant–soil regulation; for instance, in Pb+MP contexts, biochar plus melatonin improved antioxidant and hormonal regulation, reduced available Pb and oxidative damage, and partially restored biomass and grain yield (Du et al., 2026). Taken together, the available evidence indicates that co-exposure outcomes are context-dependent rather than governed by a single universal threshold. Cd responses appear closely linked to dose-dependent oxidative and cell-wall–related processes, As responses to tissue-specific regulation, Hg responses to microbial methylation-associated functionality, and Pb outcomes to the degree to which plant–soil control can be externally restored. (**Figure 1**).

**Figure 1:**
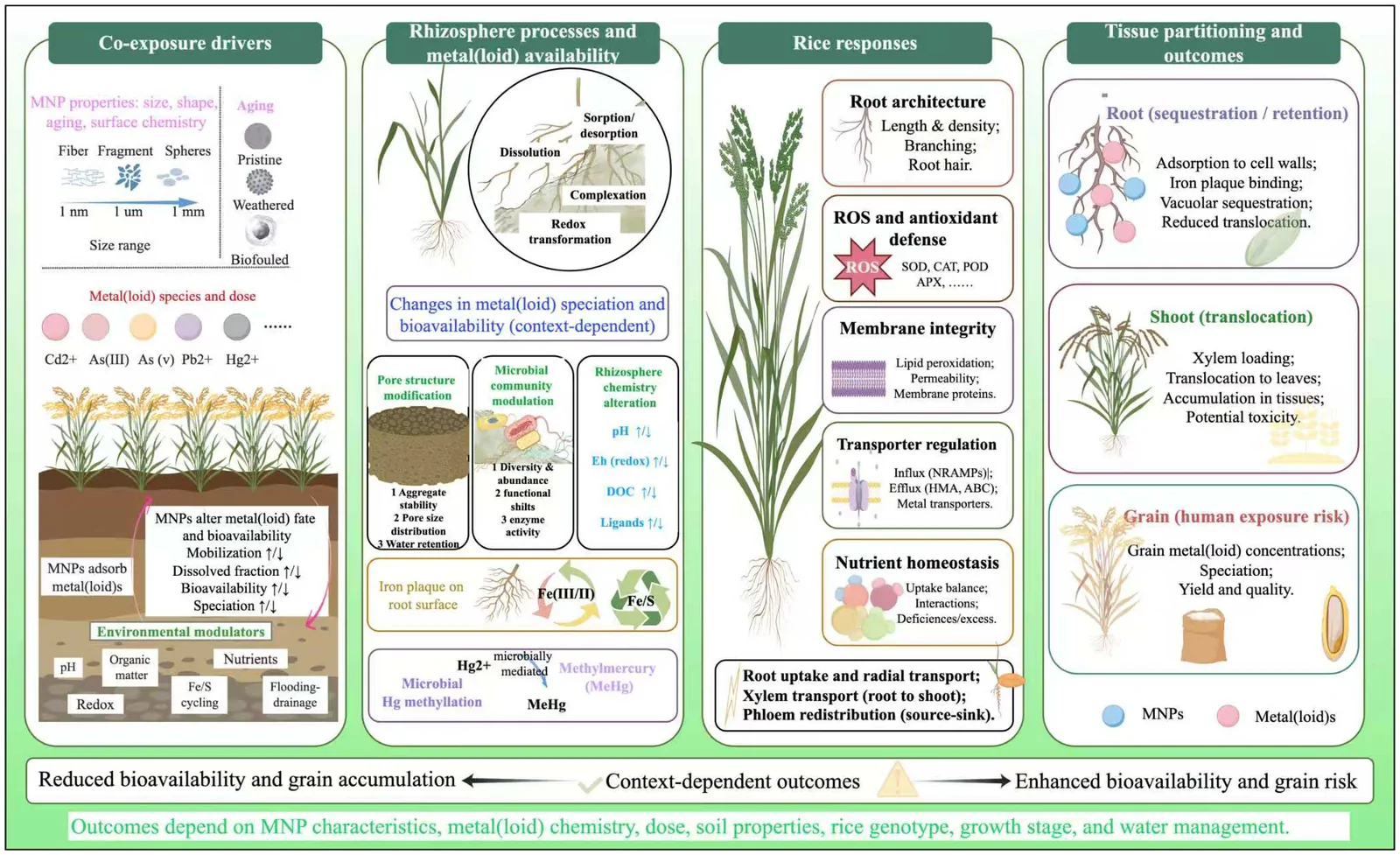
Conceptual framework for mechanisms and outcomes that vary across exposure conditions of micro/nanoplastic–metal(loid) co-exposure in rice. Micro/nanoplastic (MNP) properties, including particle size, shape, surface chemistry and aging, together with metal(loid) identity and dose, influence metal(loid) adsorption/desorption, dissolution, complexation and redox transformation in the rhizosphere. MNPs may further modify soil structure, microbial communities, pH, redox conditions, dissolved organic carbon and Fe/S cycling, thereby altering metal(loid) speciation and bioavailability. These changes affect root architecture, reactive oxygen species (ROS) balance, membrane integrity, nutrient homeostasis, root uptake, xylem transport and phloem redistribution, ultimately determining metal(loid) retention in roots, translocation to shoots and accumulation in grain. Outcomes may range from reduced metal(loid) bioavailability and grain accumulation to enhanced grain-related risk, depending on MNP characteristics, metal(loid) chemistry, exposure dose, soil properties, rice genotype, growth stage and water management. MNPs, micro/nanoplastics; Cd, cadmium; As(III), arsenite; As(V), arsenate; Pb, lead; Hg, mercury; MeHg, methylmercury; Fe, iron; S, sulfur; pH, negative logarithm of hydrogen ion activity; Eh, redox potential; DOC, dissolved organic carbon; ROS, reactive oxygen species; SOD, superoxide dismutase; CAT, catalase; POD, peroxidase; APX, ascorbate peroxidase; NRAMPs, natural resistance-associated macrophage proteins; HMAs, heavy metal ATPases; ABC, ATP-binding cassette transporters.

## 5 Integrated management strategies for reducing MNP–metal(loid) co-exposure risks in rice

In rice agroecosystems, mitigation of risks associated with MNP–metal(loid) co-exposure requires coordinated actions across source, soil, and plant–rhizosphere levels. At the system level, reducing persistent plastic inputs, improving the recovery of agricultural plastic residues, and limiting conditions that promote MNP accumulation should be precautionary priorities, given evidence that plastisphere-associated microbial communities may influence metal(loid) transformation and methylmercury-related processes under specific conditions (Hao et al., 2023; Zhu et al., 2024). At the field level, amendments and water/soil management should aim to stabilize metal(loid)s speciation and constrain Fe/S- and DOM-associated remobilization processes, because MPs can alter Fe–Mn-bound Cd, sulfate reduction, and plastisphere-associated Cd availability (Wang et al., 2024; Zhu et al., 2024). Plant–rhizosphere measures can further reduce metal(loid)s transfer to edible tissues by improving rhizosphere immobilization and plant detoxification capacity. For example, biochar plus melatonin alleviated Pb+MP stress through improved antioxidant and hormonal regulation, reduced available Pb, and modified metal(loid)s -transport-related gene expression (Du et al., 2026). Similar mitigation of MP–Cd toxicity has been reported for biochar–melatonin, silicon, and nano-Zn-modified biochar treatments (Noor et al., 2026; Yang et al., 2026). Management performance should ultimately be evaluated using mature-grain metal(loid) concentrations, relevant chemical species, yield, grain quality, soil metal(loid) speciation, and, where relevant, functional indicators of toxic-species formation such as hgcA/hgcB expression (Wenwen et al., 2025). Management should be site- and element-specific, because interventions that decrease Cd bioavailability may not necessarily reduce inorganic As accumulation. (**Figure 2**).

**Figure 2:**
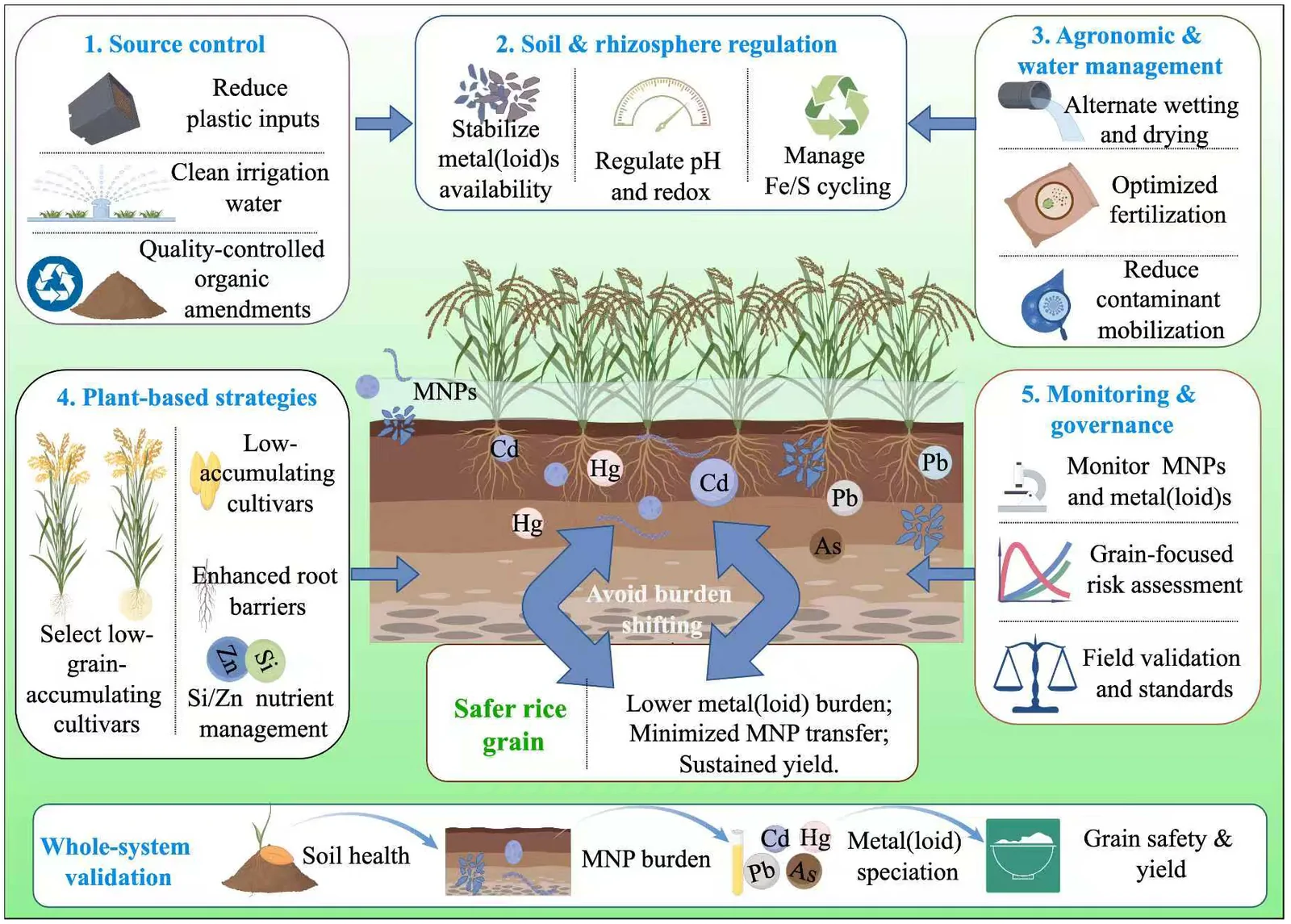
Integrated management framework for reducing micro/nanoplastic–metal(loid) co-exposure risks in rice while avoiding burden shifting. The framework integrates five complementary management domains: (1) source control through reduced plastic inputs, clean irrigation water and quality-controlled organic amendments; (2) soil and rhizosphere regulation to stabilize metal(loid) availability, regulate pH and redox conditions, and manage Fe/S cycling; (3) agronomic and water management, including site-specific alternate wetting and drying, optimized fertilization and reduced contaminant mobilization; (4) plant-based strategies, including selection of low-grain-accumulating cultivars, enhancement of root barriers and targeted silicon/zinc nutrient management; and (5) monitoring, grain-focused risk assessment, field validation and governance. Management performance should be evaluated across soil health, MNP burden, metal(loid) concentrations and speciation, grain safety, yield and grain quality to support those reductions in grain metal(loid) burden do not cause increased MNP transfer or other environmental trade-offs. MNPs, micro/nanoplastics; Fe, iron; S, sulfur; Si, silicon; Zn, zinc; Cd, cadmium; Hg, mercury; Pb, lead; As, arsenic; pH, negative logarithm of hydrogen ion activity.

### 5.1 System-level interventions to reduce co-exposure intensity and coupling triggers

System-level interventions should prioritize reducing plastic inputs and monitoring hydrological conditions that regulate metal(loid)s speciation. Under flooded conditions, MPs can increase Cd release risk by altering dissolved organic matter, Fe-mineral reduction, sulfate reduction, and the distribution of Cd among geochemical fractions (Wang et al., 2024). Plastisphere formation may further create localized microbial niches associated with Cd enrichment and Hg methylation potential (Hao et al., 2023; Zhu et al., 2024). Irrigation-water quality is also relevant because it determines As input loading, while MP type and concentration can substantially modify porewater As and exchangeable As fractions (Yu et al., 2026). Therefore, source control, irrigation-water screening, and field water management are reasonable precautionary strategies; however, their effectiveness in suppressing MNP-mediated metal(loid)s transformations remains to be tested in long-term field experiments. For Hg-contaminated systems, monitoring should include MeHg and methylation indicators, such as hgcA/hgcB, in addition to total Hg, because MPs may alter Hg methylation through plastisphere-associated microbial communities and Fe/S- and DOM-related processes (Yang et al., 2022; Hao et al., 2023; Wenwen et al., 2025).

### 5.2 Field-scale practices for stabilizing metal(loid)s bioavailability and disrupting plastics-mediated transformation

At the field scale, amendments can be used to reduce metal(loid)s mobility and strengthen rhizosphere retention, although their ability to counteract MNP-mediated transformations requires direct testing. In Cd–As co-contaminated paddy soil, goethite-modified biochar increased relatively immobile metal(loid)s fractions, enhanced Fe accumulation in root plaques, and reduced Cd and As accumulation in rice grain (Irshad et al., 2022). These functions are relevant to MNP-contaminated soils because MPs can alter Cd availability through DOM-, Fe-mineral-, sulfate-reduction-, and plastisphere-associated processes (Wang et al., 2024; Zhu et al., 2024). Under Pb+MP stress, combined biochar and melatonin improved biomass and grain yield, reduced available Pb and oxidative damage, and altered metal(loid)s -transport-related gene expression (Du et al., 2026). For As, NPs reduced root iron-plaque formation by 22.5% and coincided with increased As accumulation and translocation, indicating that maintaining Fe-associated rhizoplane retention is a plausible management target (Jiang Y. et al., 2024). Hg management should additionally assess MeHg formation and methylation-related microbial functions, rather than total Hg alone: PLA MPs increased soil Hg methylation potential and grain Hg reached 130.46 μg kg−1, approximately sixfold above the control (Wenwen et al., 2025). Plastisphere-associated MeHg production provides further support for including functional transformation indicators in field-risk assessment (Hao et al., 2023). Evidence from an Sb–MP system further supports the multifunctional potential of engineered biochar. In rice pots co-contaminated with Sb and MPs, micron-engineered biochar reduced soil and plant Sb by 31%, improved nutrient status and grain yield, and reshaped bacterial communities and stress- and transport-related transcriptomic responses (Hassan et al., 2026). Although Sb is outside the main metal(loid)s considered here, this study suggests that amendment performance should be evaluated across soil immobilization, microbiome function, plant regulation, and grain endpoints.

### 5.3 Plant and rhizosphere strategies to limit metal(loid)s entry flux, reconfigure detoxification, and shift tissue partitioning

Plant–rhizosphere interventions can aim to reduce metal(loid)s accumulation in rice by strengthening rhizosphere retention and plant detoxification capacity. In As-exposed rice, NPs reduced root iron-plaque formation by 22.5%, decreased ABA, and upregulated aquaporin-related genes, coinciding with increased As accumulation and translocation (Jiang Y. et al., 2024). These findings identify maintenance of Fe-associated rhizoplane retention and stress regulation as plausible intervention targets. Polymer-specific Cd responses further indicate that management should consider rhizosphere chemical and plant physiological processes together. PET lowered rhizosphere pH from 7.05 to 6.30, accompanied by remobilization of mineral-bound Cd and an increase in brown-rice Cd from 0.03 to 0.06 mg kg^−1^, whereas PVC induced strong root oxidative stress (MDA + 78%; POD + 60%) (Zhou et al., 2026). At high NP concentration, NP–Cd co-exposure was associated with greater root-growth inhibition than Cd exposure alone, with root length, root-tip number, and root surface area decreasing by 14%, 22%, and 4%, respectively (Liu et al., 2026). Amendments that jointly improve soil conditions and plant stress responses show mitigation potential. BC+MT alleviated Pb+MP stress through changes in antioxidant activity, hormonal regulation, available Pb, and metal(loid)s -transport-related gene expression (Du et al., 2026). Under MP–Cd co-exposure, BC+MT also reduced Cd bioavailability and rice Cd accumulation while enhancing antioxidant responses and downregulating Cd-transport-related genes (Yang et al., 2026). Beyond engineered amendments, Zn-based nutritional regulation may improve rice tolerance to MNP stress through both metabolic and physical mechanisms. Under PS exposure, ZnSO_4_ partly restored seedling growth by modulating shoot nitrogen metabolism and root carbon metabolism while reducing oxidative stress (Liu et al., 2025). Zn supplementation with IAA further alleviated injury caused by positively charged PS nanoplastics, accompanied by reduced particle distribution and improved redox homeostasis (Xu N. et al., 2023). In fragrant rice, optimized nZnO also reduced root-associated nanoplastic uptake and partially restored NP-disrupted 2-AP-related metabolism (Imran et al., 2024). However, these seedling-based findings do not yet establish protection of grain quality, long-term safety of Zn-based inputs, or reduced Cd, As, Hg, and Pb accumulation.

### 5.4 Outcome-based validation using root–shoot–grain endpoints to assess food-safety effectiveness

Management effectiveness should ultimately be evaluated using mature-grain food-safety endpoints rather than inferred from a single soil or root indicator. A multi-level assessment can integrate rhizoplane retention indicators (e.g., iron plaque and bioavailable metal(loid) fractions), plant physiological and molecular responses (e.g., oxidative damage, hormone status, and metal(loid)-transport- or chelation-related genes), and metal(loid) concentrations in roots, shoots, and mature grain. This integrated design is important because responses to NP–metal(loid) co-exposure can differ among plant organs. Under NP–As(III) co-exposure, transcriptional responses differed between roots and leaves; the original study reported relatively additive expression patterns in roots and more complex non-additive expression patterns in leaves under its analytical framework (Xu, 2026). However, these molecular expression patterns should not be interpreted as direct evidence of interaction effects on metal(loid) uptake, grain accumulation, or food-safety risk. Speciation-sensitive endpoints are particularly important for Hg and As. PLA MPs were associated with increased soil Hg methylation potential; in the same study, total Hg concentration in grain reached 130.46 μg kg^-1^, approximately sixfold higher than in the control. Therefore, total Hg measurements should, where possible, be complemented by MeHg concentrations and methylation-related indicators (Hao et al., 2023; Wenwen et al., 2025). For As, NPs reduced root iron plaque by 22.5% and altered ABA-, aquaporin-, and glutathione-related responses, coinciding with increased As accumulation and translocation (Jiang Y. et al., 2024). For Cd, PET-associated rhizosphere acidification and Cd remobilization were accompanied by increased brown-rice Cd concentrations (Zhou et al., 2026).

Together, root–shoot–grain metal(loid) partitioning, relevant chemical species, functional indicators, yield, grain quality, and MNP abundance and characteristics provide a more robust basis for judging whether an intervention reduces metal(loid) concentrations and food-safety risks in edible rice grain. Management interventions should not be judged solely by reductions in soil-extractable metal(loid)s or vegetative-tissue concentrations. Their effectiveness should be validated using mature-grain metal(loid) concentrations, relevant chemical species, yield and grain quality, MNP abundance and characteristics, and potential secondary environmental effects, including changes in redox-sensitive element cycling and methylmercury formation.

### 5.5 Integrated management: comparative evaluation of advantages, limitations, and uncertainty

To move beyond a catalogue of remediation options, integrated management should be evaluated by comparing likely advantages against key limitations and uncertainty, particularly for MNP–metal(loid) co-exposure outcomes and mature-grain food-safety endpoints (Wang et al., 2024; Zhu et al., 2024). Biochar can immobilize metal(loid)s through adsorption/complexation and can modify rhizosphere chemistry; however, performance is highly feedstock- and aging-dependent, and changes in dissolved organic matter and redox conditions may non-uniformly alter metal(loid) speciation and potentially shift rather than eliminate bioavailability (Irshad et al., 2022). Silicon may enhance plant tolerance and strengthen physical/physiological barriers that indirectly reduce metal(loid) transfer to edible tissues; nevertheless, its effectiveness can be element-specific and may not reliably address speciation-sensitive risks (e.g., for As and Hg) (Noor et al., 2026). Melatonin primarily operates as a plant regulatory/tolerance-promoting compound, offering antioxidant and hormonal mitigation; yet evidence for consistent reductions in metal(loid) concentrations in mature grains and for controlling MNP-mediated chemical transformations remains limited (Du et al., 2026). Broad soil amendments (e.g., pH/redox and Fe/S-DOM related regulators) can target coupled geochemical processes that govern metal(loid) remobilization; but trade-offs (including Cd–As antagonism/activation) and secondary effects on microbial functions (relevant to Hg methylation) require element- and condition-specific verification (Hao et al., 2023; Wang et al., 2024). Finally, agronomic management (e.g., water regime, irrigation quality, and optimized fertilization) is often more scalable and can directly modulate redox status and metal(loid) mobility; however, its impact is strongly site-dependent and must be validated to avoid burden shifting, given that MNP redistribution may alter metal(loid)–rhizosphere coupling across growth stages (Yu et al., 2026). Accordingly, interventions should be judged not only by reductions in soil-extractable metal(loid)s or vegetative-tissue levels, but also by direct confirmation using mature-grain concentrations and relevant chemical species, together with indicators of MNP burden/characteristics and potential secondary environmental outcomes (Authority, 2009).

## 6 Conclusion and future perspectives

MNPs and priority food-safety-relevant metal(loid)s, including Cd, As, Hg/MeHg, and Pb, frequently co-occur in rice agroecosystems, jointly influencing rice performance and food-safety-relevant endpoints under realistic multi-contaminant conditions. Evidence across soil, rhizosphere, and plant scales indicates that co-exposure outcomes cannot always be predicted from single-contaminant studies and are shaped by coupled changes in physicochemical conditions, microbial processes, and plant responses. MNPs can alter metal(loid) transformation, speciation, and bioavailability by modifying soil and rhizosphere conditions, including pH, redox status, dissolved organic matter, microbial activity, and iron-plaque-associated retention. These changes may subsequently affect root entry and tissue partitioning. As a result, Cd, As, Hg, and Pb may show directionally different responses in bioavailability, chemical transformation, tissue accumulation, and plant-performance endpoints. However, the amount and quality of evidence differ substantially among elements, with the strongest evidence currently available for Cd and As and more limited rice-specific evidence for Hg and Pb.

Based on current evidence, risk mitigation should integrate source control, site-specific soil and water management, and plant-centred strategies. At the system scale, measures should reduce plastic inputs and metal(loid) emissions from irrigation water, sludge, fertilizers, and other agricultural sources. At the field scale, management should be tailored to soil properties and target elements, with particular attention to pH, redox conditions, dissolved organic matter, iron cycling, and potential Cd–As trade-offs. At the plant–rhizosphere scale, suitable cultivars, balanced nutrient management, and carefully evaluated amendments may help reduce metal(loid) bioavailability and metal(loid) concentrations in mature rice grain. Effectiveness should be verified using mature-grain metal(loid) concentrations, relevant chemical species, yield, grain quality, and potential secondary environmental burdens. Future research priorities should be made more specific, with emphasis on long-term field experiments that capture multi-season accumulation, soil legacy effects, and realistic variability in co-exposure; standardized exposure protocols for MNP characteristics (e.g., size, polymer type, and aging/weathering state) alongside consistent metal(loid) concentration reporting to improve comparability across studies; multi-omics validation (e.g., transcriptomics/proteomics/metabolomics integrated with microbiome profiling) to confirm mechanistic links between MNP-driven environmental changes and metal(loid) uptake and transformations; grain speciation and bioaccessibility measurements using food-relevant extraction and ingestion-based approaches rather than relying solely on bulk concentrations; and climate change interaction studies (e.g., temperature, rainfall/runoff patterns, and altered redox cycling) to clarify how warming and hydrological shifts modify MNP–metal co-contamination behavior and rice food-safety outcomes.
