# Supplementary material for: Micro/nanoplastics–metal(loid) co-exposure in rice: evidence, mechanisms, and food-safety implications
Source: Front Plant Sci. 2026 Oct 20;17:1963280. doi: 10.3389/fpls.2026.1963280 (PMC13728495; doi:10.3389/fpls.2026.1963280)
Supplement: Supplementary file 1 [file SupplementaryFile1.docx]

**Supplementary Figure 1 Literature search and screening flow.**
